# Management of Primary Uterine Cervix B-Cell Lymphoma Stage IE and Fertility Sparing Outcome: A Systematic Review of the Literature

**DOI:** 10.3390/cancers15143679

**Published:** 2023-07-19

**Authors:** Guglielmo Stabile, Chiara Ripepi, Lara Sancin, Stefano Restaino, Francesco Paolo Mangino, Luigi Nappi, Giuseppe Ricci

**Affiliations:** 1 Institute for Maternal and Child Health, IRCCS “Burlo Garofolo”, Via dell’Istria 65/1, 34137 Trieste, Italy; francesco.mangino@burlo.trieste.it (F.P.M.); giuseppe.ricci@burlo.trieste.it (G.R.); 2 UCO Clinica Ostetrica e Ginecologica, Department of Medicine, Surgery and Health Sciences, University of Trieste, 34137 Trieste, Italy; chiara.ripepi@burlo.trieste.it (C.R.); lara.sancin@burlo.trieste.it (L.S.); 3 Obstetrics and Gynecology Unit, Department of Obstetrics, Gynecology and Pediatrics, Department of Medical Area DAME, Udine University Hospital, 33100 Udine, Italy; restaino.stefano@gmail.com; 4 Departments of Obstetrics and Gynecology and Medical and Surgical Sciences, University of Foggia, 71122 Foggia, Italy; luigi.nappi@unifg.it

**Keywords:** cervical lymphoma, management, therapy, fertility sparing, pregnancy outcome

## Abstract

**Simple Summary:**

No guidelines regarding optimal treatment exist for primary cervical lymphomas. We have performed a systematic review of the literature about the management of this rare pathology. Conservative treatment with the combination of surgery and chemotherapy or surgery and radiotherapy has been reported in a few cases with apparent success. Furthermore, we have reported pregnancy outcome in patients treated with a fertility sparing approach.

**Abstract:**

The female genital tract can be involved as a secondary manifestation of disseminated lymphomas or leukaemia but can rarely be the primary site of so-called extranodal lymphomas. Primary lymphomas of the female genital tract can affect the uterine corpus, uterine cervix, vulva, vagina, or adnexa. Only about 0.008% of all cervical tumours are primary malignant lymphomas. The most common clinical presentation of primary cervical lymphomas is a history of prolonged minor abnormal uterine bleeding, while unstoppable bleeding at presentation is rarely reported in the literature. “B” symptoms related to nodal lymphomas are usually absent. Since vaginal bleeding is a nonspecific symptom, the first diagnostic hypothesis is usually of one of the more common female genital conditions such as cervical or endometrial carcinoma or sarcoma, fibroids, adenomyosis, or endometriosis. Cervical cytology is usually negative. Preoperative diagnosis requires deep cervical biopsy. No guidelines regarding optimal treatment exists; radiotherapy, chemotherapy, and surgery are used in different combinations. Conservative treatment with the combination of surgery and chemotherapy or surgery and radiotherapy has been reported in a few cases with apparent success. With this review, we aim to understand what the best therapeutic approaches for this rare pathology in young and elderly women are. Moreover, we find favorable pregnancy outcome in patients treated with a fertility sparing approach.

## 1. Introduction

The female genital tract can be involved as a secondary manifestation of disseminated lymphomas or leukaemia, but rarely is the primary site of so-called extranodal lymphomas. Primary lymphomas of the female genital tract can affect the uterine corpus, uterine cervix, vulva, vagina, or adnexa [1].

From the early 1970s to the early 21st century, the incidence rates of non-Hodgkin lymphoma (NHL) nearly doubled in the United States. Although this increase can be partially explained as the effect of earlier detection resulting from improved diagnostic techniques, the rise remains mostly unexplained [2]. Currently, NHLs account for around 4% of all new cancer diagnoses [3]. Approximately one third are extranodal and can affect the gastrointestinal tract, breast, CNS, mediastinum, bone, female genital tract or testis, thyroid, skin, oropharynx, spleen, liver, and many other organs. Around 0.5–1.5% of all extranodal NHLs arise from the female genital tract and only 0.12–0.6% from the cervix. Nevertheless, only about 0.008% of all cervical tumours are primary malignant lymphomas [4,5,6,7,8,9,10,11].

In the early stages, it is quite easy to define it as a primary cervical lymphoma since it is confined to the cervix. The strictest definition of primary extranodal NHL is that of a lymphoma presenting only in extranodal sites, with no visible lymphadenopathy on imaging; thus, only I E lymphomas are included (Ann Arbor staging) [12]. Moreover, when the disease involves contiguous sites (e.g., cervix and uterine corpus or cervix and upper vagina), the site with the largest area of involvement is defined as the primary site [13]. The tumour must be confined to regional lymph nodes or neighbouring organs at diagnosis, with no bone marrow involvement, a lack of malignant cells in peripheral blood, and any distant disease must occur at least several months after the appearance of primary lesions [14,15,16].

The history of prolonged minor abnormal uterine bleeding is the most common presenting symptom of primary cervical lymphoma. Heavy bleeding at presentation is rare with only a few cases reported in the literature [17,18,19]. “B” symptoms such as fever, fatigue, weight loss, and night sweats are usually absent. Since postmenopausal vaginal bleeding or intermenstrual bleeding is a nonspecific symptom, the first consideration would be on one of the more common female genital tract pathologies [20]. A diagnostic pitfall to consider is that cervical cytology is most of the time negative. This reflects the peculiar behaviour of lymphomas which usually spread through cervical stroma leaving the epithelium intact at least in the early stages, differently from cervical adenocarcinoma. Therefore, preoperative diagnosis requires deep cervical biopsy [9]. The most frequent histological subtype is diffuse large B-cell lymphoma (DLBCL) accounting for more than 70% of all cases [21,22].

Moreover, fertility-sparing approach is increasing in the treatment of cancer in young women with satisfying overall survival end point. Even in cervical lymphoma, it has been described with a conservative approach avoiding RT and invasive surgery with optimal results.

To the best of the authors’ knowledge, the literature about primary lymphomas of the uterine cervix is limited to single case reports and case series. There are no greater studies or randomized controlled trials regarding diagnostic algorithm or treatment strategy. To date, unanimous consensus or published guidelines for the management of primary lymphomas of the uterine cervix do not exist.

We aim to analyze and group the different treatment options presented in the literature to try to provide a general common point of view and understand what the best therapeutic approaches for this rare pathology are. Particularly, we try to focus on the conservative management in fertile women who desire to preserve their fertility and we want to analyze the outcome of pregnancies following treatment.

## 2. Materials and Methods

This retrospective observational descriptive study was approved by our institutional review board (IRB/Burlo RC August 2020). Bibliographic search was conducted on Medline using PubMed, Scopus, and Web of Science. The aim was to find articles regarding primary diffuse large B-cell lymphoma of the uterine cervix. The search terms used were “lymphoma”, “cervix”, “B-cell”, and “Cervical cancer” with filters applied to display only articles in English from year 1995 up to 1 October 2022. A total of 166 articles were identified through Pubmed, 268 through Scopus database, and 35 through Web of Science. We decided to collect only articles published from 1995 in order to have sufficiently homogeneous data about histologic classification; in fact, at the end of 1994, the new REAL classification of lymphomas was published, and that revolutionary classification is still the base of the successive WHO classifications published in 2001, 2008, 2016, and updated again in 2017 [23,24,25,26].

We identified 471 records through databases search (n = 167 from PubMed; n = 269 from Scopus; and n = 35 from Web of Science). After removing 185 duplicate records, 286 remained to be screened. After screening, 262 records were excluded as they did not meet inclusion criteria, because they were not relevant, or for other reasons (articles with unclassified or unclear stage or unclear histologic subtype). At the end of the screening process, we included in our review thirty-four studies for a total of forty-six clinical cases.

**Figure 1:**
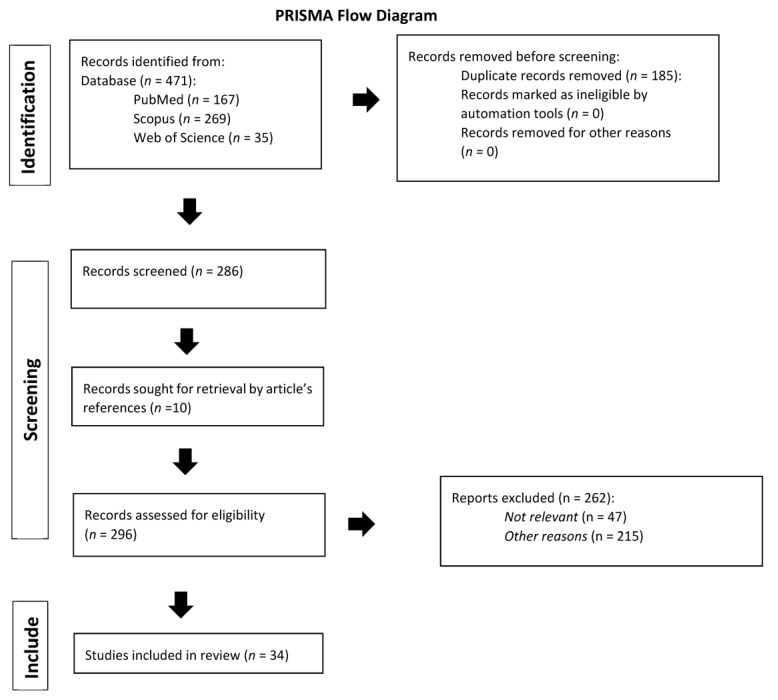
Prisma flow diagram [24].

All articles were listed by title, author, and year of publication. Following the PRISMA checklist [Figure 1], three (L.S., G.S., C.R.) independent investigators screened all the articles by title and abstract to identify those eligible [27]. Titles and abstracts were screened identifying potentially relevant articles that have been subsequently reviewed. Articles selection was concluded on 1 October 2022. Inclusion criteria were cases of primary extranodal diffuse large B-cell lymphomas arising from the uterine cervix, classified as stage IE (Ann Arbor staging), and English-language articles. We evaluated all types of articles (original article, video article, case report, review article, and metanalysis) resulting from our search on databases. Exclusion criteria were primary extra-cervical gynaecological lymphomas extending secondarily to the cervix, secondary involvement of the cervix in leukaemia or primary nodal disseminated lymphomas, cervical relapse of previously diagnosed lymphoma/leukaemia, advanced disease (stage IIE, IIIE or IVE), cervical disease in HIV-positive patients, transplanted patients or patients using immunosuppressive therapy for any reason, patients with a previous diagnosis of tumor, articles published before year 1995, articles with unclassified stage or unclear histologic subtype, and articles not written in English. The systematic review was not submitted to Prospero as only a limited number of case reports were found in the literature [28]. Three authors (L.S., G.S., C.R.) reviewed independently all identified full text papers, selecting those that met predefined eligibility criteria. Discrepancies were resolved by consensus. Another 10 articles were identified through references of the previously selected papers. The methodological quality of the included studies was assessed using the JBI Critical Appraisal Checklist for case reports [Table 1] and for case series [Table 2, Appendix A].

## 3. Results

At the end of the systematic review, we included in our study thirty-four studies for a total of forty-six clinical cases [Table 3].

In our analysis, the median age of women affected by primary lymphoma of the cervix uteri were 50.0 years (range 20–85 years). Regarding the age range at diagnosis, 28.2% (13/46) were under 40 years, 47.8% (22/46) were 40–60 years old, and 23.9% (11/46) were over 60 years old. More than half of the patients were known to be in post-menopause at the time of diagnosis (24/46, 52.2%), 36.9% (g) of patients were in pre-menopause, and of the remaining 5 patients (10.8%) we have no data on this.

**Figure 2:**
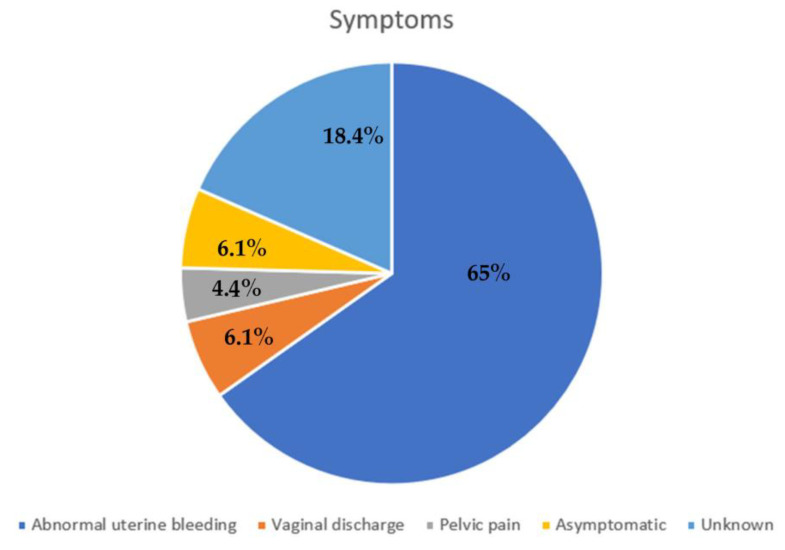
Most frequent symptoms.

The most frequent symptoms manifested by patients at the time of diagnosis were abnormal uterine bleeding (65%), vaginal discharge (6.1%), and pelvic pain (4.4%). A total of 6.1% of patients were asymptomatic and of 18.4% we have no data on this [Figure 2]. Referring to systemic symptoms, 88% of the patients did not present the typical “B symptoms” of lymphomas, such as mild-fever, fatigue, weight loss, and night sweats. Only one patient (2%) presented weight loss.

The median size of the mass at diagnosis was 6.6 cm, with a minimum diameter of 2 cm and a maximum of 11 cm.

The therapeutic approach was varied. The different approaches were as follows: CT + RT (36.9%), CT only (21.7%), surgery + adjuvant CT (10.9%), surgery + adjuvant RT (6.5%), neoadjuvant CT + surgery (6.5%), surgery only (4.3%), neoadjuvant CT + conization + CT (2.2%) and surgery + adjuvant CT/RT (2.2%), CT + ovarian suppression after cryopreservation (2.2%), CT + ovarian suppression (2.2%), and CT + ovarian transposition + RT (2.2%). We published the only case completely treated with a hysteroscopic approach followed by adjuvant RT.

After treatment, patients started a follow-up program. After 12 months from diagnosis, 93.5% of patients were still disease free, 2.2% had had a partial response to therapy, 2.2% relapsed, and only one patient was dead (2.2%). After 5 years from diagnosis, considering only those patients we had data about (19/45), 84% were still disease free, 6.3% relapsed, 6.3% had had a partial response, and only one patient was dead (6.3%). We have no data about the follow-up of three patients.

Even if only 28.2% of case reports are about women under 40 years old, only 5 papers reported pregnancy data. Of this, only one was spontaneously miscarried [36], while the others have reached the full term with no complications during pregnancy. Regarding the mode of delivery, 3/4 (75%) had a vaginal delivery without any complication, while 1/4 had a planned Caesarean section at 38 gestational weeks [53]. Only in 1/4 cases was the labour inducted for post-term pregnancy with Prostaglandin E2 pessary. The mean time for attempting pregnancy was 33 months after treatment (range 12–66 months). The treatment approach was different between these five women, but all of them underwent CT (CHOP ± Rituximab) [Table 4].

## 4. Discussion

### 4.1. Symptoms and Diagnosis

Primary cervix uteri lymphoma is a rare pathology; therefore, gynecologists should be aware when evaluating cervical lesions. Isolated and persistent vaginal bleeding without pain and without any “B symptom” should increase the level of suspicion. PAP test is usually negative, and a deep biopsy is needed to obtain a conclusive diagnosis.

The presence of a diffusely enlarged cervix or a cervical mass should trigger further investigation such as transvaginal echography and a computed tomography or magnetic resonance to better define the lesion and its extension both locally and eventually out of the pelvic. This kind of lesion can easily mimic cervical adenocarcinoma or sarcoma, but also benign conditions like fibroids, especially colliquated fibroids.

Characterization of the tumour immunophenotype is nowadays mandatory to achieve a precise diagnosis and should be made according to the latest WHO classification. Primary cervical lymphomas are usually B-cell type NHL, but there are some cases of primary cervical Hodgkin lymphoma, MALToma, and T-cell lymphoma described. The most common subtype is diffuse large B-cell lymphoma, accounting for around 70% of the cases. Older case reports do not follow the WHO classification for the characterization of tumour histology, but most reported cases are B-cell type lymphomas.

### 4.2. Therapy

Neither clinical trials nor international guidelines exist due to the rarity of this disease. Therefore, there is no consensus on what the best management of this disease is. In general, the cornerstone of lymphoma treatment is chemotherapy, so most reported cases are treated accordingly with chemotherapy alone or in association with radiotherapy.

RT plays a pivotal role as a treatment tool for this rare disease, as we know from literature data of other site primary extranodal NHL [58]. In fact, RT has a local effect avoiding systemic complications and can help to control symptoms in those women who are not able to address CHT or surgery. Surgery (mainly total abdominal hysterectomy with or without salpingo/oophorectomy and lymphadenectomy) is usually performed cautiously if a precise preoperative diagnosis is not possible. In a published manuscript, we have reported a case treated with hysteroscopic resection, which to date is the only one reported in the literature [56]. Hysteroscopy appears to be a very promising tool for the treatment of organ-limited utero-cervical pathology [29,30]. Rather, some reported cases of massive bleeding due to cervical lymphoma that was managed with emergent embolization of uterine arteries or emergent hysterectomy exists. Considering the age range at diagnosis of this disease, it is important to find out a conservative approach.

### 4.3. Fertility Sparing Treatment

To date, in young women who want to preserve their fertility, the approach has been chemotherapy (CHOP cyclophosphamide-doxorubicin-vincristine prednisone chemotherapy regime) and immunotherapy to reduce the need for radiotherapy or surgical resection. To the best of our knowledge, to date, there are no reported cases in the literature treated with cold knife or trachelectomy alone. In this review, when a fertility approach is sought, chemotherapy alone is the treatment of choice [33,37,38,46], followed by CT + ovarian transposition + RT and CT + cold knife conization [45,53]. The reported evidence regarding non-Hodgkin lymphoma in general suggests that in cases of childhood NHL, and in cases where fertility preservation is desired, current chemotherapeutic regimens are safe and can spare fertility, particularly when GnRH agonists are used in conjunction with treatment. As reported by Quaresima et al., who injected Leuprolide 11.25 mg im before therapy every 12 weeks with successful outcome [29]. Signorelli et al. reported a case series of cervical lymphoma treated with a conservative approach (CT ± surgery) and observed that almost all women resumed menses within 5 months after the end of their treatment, and only one needed hormone replacement treatment [46]. Other fertility-spearing techniques exist as ovarian transposition in those women who undergo to RT [43].

### 4.4. Pregnancy Outcome after Treatment

In patients who become pregnant after cervix uteri lymphoma, mode of delivery should be well discussed before childbirth, particularly if surgery or RT has been performed, even if RT does not seem to reduce the possibility of a safe vaginal delivery. In our review, 75% of pregnant patients had a vaginal delivery without any complications. This is a fact that encourages vaginal delivery in these patients regardless of the type of therapy performed.

Ferreri et al. reported a case of vaginal delivery 36 months after pelvic irradiation, without any complication during and after the delivery [45]. Parva et al. and Quaresima et al. reported two cases of safe vaginal delivery after chemotherapeutic treatment, without complication neither during pregnancy nor during the delivery [29,43]. Whereas Lorusso et al. reported a case of a planned Cesarean section at 38 gestational weeks 3 years after completing the primary treatment (R-CHOP + cold knife conization) [53].

Clearly, there are no guidelines regarding the safest mode of delivery for these patients. However, the few data available suggest that in carefully selected patients with fully treated non-Hodgkin lymphoma of the cervix with no apparent disease, it may be plausible and even prudent to allow a trial of labour with some likelihood of a successful vaginal delivery [43].

In light of our systematic review, in young women considering future pregnancy, the fertility sparing surgery as cold knife conization, trachelectomy, or hysteroscopic resection could be feasible considering the indolent course of the disease even if there are few data available and all are from case reports and case series. Moreover, Perren et al., following an extensive revision of the literature, concluded that there is no evidence that radical surgery confers more survival advantages than conservative surgery in patients with localized and low- or intermediate-grade tumors [59].

From a recent literature review by Capsa et al. emerges that the combination of CHOP CT regimen and RT as adjuvant is the most frequently chosen by clinicians with optimal results on prognosis [31].

Nevertheless, from the data of this review emerges a non-inferiority of the surgery + RT approach (considering ovarian transposition) compared to the CT ± RT approach. Furthermore, also in elderly women or in patients who are ineligible for chemotherapy, a conservative surgery and radiotherapic approach can be considered in order to reduce side effects and morbidity [56]. In conclusion, in the absence of a consensus on the best management, proper instrumental staging and detailed counselling with the patient is recommended to tailor the best treatment.

## 5. Conclusions

Primary cervical lymphoma is a very rare entity and affects a wide range of ages. Usually, in the early stages, primary cervical lymphoma has an excellent prognosis with any treatment combination (radiotherapy and/or chemotherapy and/or surgical resection). Considering data of follow-ups, in most cases, those patients who do not receive local therapy with RT have an excellent prognosis and this can suggest avoiding it in those women with childbearing desire. Moreover, in these cases, RT will become a tool in case of relapse. There is evidently the need for a larger study, possibly a multicentric randomized controlled trial, to define a specific classification and the best management for this rare tumour, even if due to the rarity of this pathology it would not be feasible. Particularly, it is important to look for an approach as conservative as possible considering that 30% of diagnosis occurs in women under 40 years old. Moreover, when a conservative approach is performed, we should ask if definitive surgery is necessary or recommended at the end of childbearing desire.

## Figures and Tables

**Table 1:** JBI Critical Appraisal Checklist for case reports (D1–D8 represent the eight questions about each case report).

Author, Year	D1	D2	D3	D4	D5	D6	D7	D8
Stabile et al., 2022 [27]	Yes	Yes	Yes	Yes	Yes	Yes	Yes	Yes
Quaresima et al., 2022 [28]	Yes	Yes	Yes	Yes	Yes	Yes	Yes	Yes
Capsa et al., 2022 [29]	Yes	Yes	Yes	Yes	Yes	Yes	Unclear	Yes
Akkour et al., 2021 [30]	Yes	Yes	Yes	Yes	Yes	Yes	Yes	Yes
Gui et al., 2019 [31]	Yes	Yes	Unclear	Yes	Yes	Yes	Unclear	Yes
Boussios et al., 2018 [32]	Unclear	No	Unclear	No	Yes	No	Unclear	Yes
Roberts et al., 2018 [33]	Yes	Yes	Yes	Yes	Yes	Yes	Yes	Yes
Cubo et al., 2017 [34]	Yes	Yes	Yes	Yes	Yes	Yes	Unclear	Yes
Fratoni et al., 2016 [35]	Yes	Yes	Yes	Yes	Yes	Yes	Yes	Yes
Posfai et al., 2015 [36]	Yes	Unclear	Yes	Yes	Yes	Unclear	Unclear	Yes
Mouhajir et al., 2014 [37]	Yes	No	Yes	Unclear	Yes	Yes	No	Yes
Binesh et al., 2012 [38]	Yes	Yes	Yes	Yes	Yes	Yes	Yes	Yes
Parnis et al., 2012 [39]	Yes	Yes	Yes	Yes	Yes	Yes	Unclear	Yes
Vasudev et al., 2012 [40]	Yes	No	Yes	Yes	Yes	Yes	Unclear	Yes
Parva et al., 2011 [41]	Yes	Yes	Yes	Yes	Yes	Yes	Yes	Yes
Upanal et al., 2011 [42]	Yes	No	Yes	Yes	Yes	Yes	Unclear	Yes
Baijal et al., 2009 [3]	Yes	No	Yes	Unclear	Yes	Yes	Unclear	No
Ab Hamid et al., 2008 [7]	Yes	Yes	Yes	Yes	Yes	Yes	Yes	Yes
Ferreri et al., 2008 [43]	Yes	Yes	Yes	Yes	Yes	Yes	Yes	Yes
Wuntakal et al., 2008 [44]	Yes	Unclear	Yes	Yes	Yes	Yes	Unclear	Yes
Lorusso et al., 2007 [45]	Yes	Yes	Yes	Yes	Yes	Yes	Yes	Yes
Semczuk et al., 2006 [46]	Yes	Yes	Yes	Yes	Yes	Yes	Unclear	Yes
Gonzalez-Cejudo et al., 2006 [47]	Unclear	No	Unclear	No	Yes	No	Unclear	Unclear
Dursun et al., 2005 [48]	Yes	No	Yes	Yes	Yes	Yes	Unclear	Yes
Chandy et al., 1998 [49]	Yes	Yes	Yes	Yes	Yes	Yes	Yes	Yes
Nasu et al., 1998 [50]	Yes	No	Unclear	No	Yes	No	Unclear	Unclear

**Table 2:** JBI Critical Appraisal Checklist for case series (D1–D10 represent the ten questions about each case series).

Author, Year	D1	D2	D3	D4	D5	D6	D7	D8	D9	D10
Goda et al., 2020 [18]	Yes	Yes	Unclear	Unclear	Yes	Yes	Yes	Yes	Yes	Unclear
Cao et al., 2014 [51]	Yes	Yes	Unclear	Unclear	Yes	Yes	Yes	Yes	Yes	Unclear
Signorelli et al., 2007 [52]	Yes	Yes	Yes	Yes	Yes	Yes	Yes	Yes	Yes	Unclear
Hariprasad et al., 2006 [53]	Yes	Yes	Unclear	No	Yes	Yes	Yes	Yes	Yes	Unclear
Chan et al., 2005 [19]	Yes	Yes	Unclear	No	Yes	Yes	Yes	Yes	Yes	Unclear
Lee et al., 1998 [54]	Yes	Yes	Unclear	No	Yes	Yes	Yes	Yes	Yes	Unclear
Makarewicz et al., 1995 [55]	Yes	Yes	Unclear	No	Yes	Yes	Yes	Yes	Yes	Unclear
Stroh et al., 1995 [8]	Yes	Yes	Yes	Yes	Yes	Yes	Yes	Yes	Yes	Unclear

**Table 3:** Case identified after systematic review.

Age	Menopause	Presence of “B” Symptoms	DLBCL Subtype	Immuno-Phenotype	Macroscopic Appearance /Dimension	PAP Smear	Diagnosis	Treatment	Outcome/Follow-Up (Months)	Reference
83	Yes	No	GCB		Cervical mass/4 cm	-	Surgical specimen	Hysteroscopic resection + RT ()	DF/	Stabile et al., 2022 [28]
30	No	No	-	CD20+, CD10+, BCL6+, PAX5+	Cervical mass/5 cm	Normal	Hysteroscopi c biopsy and D&C	CT (R-CHOP ×8) + ovarian suppression	DF/-	Quaresima et al., 2022 [56]
75	Yes	No	-	CD20+, CD10-, CD23-, CD3-, CD5-, Ki 67 50%	Cervical mass extended to upper vagina/4 cm	-	Biopsy	CT (CHOP) + RT (45 Gy)	DF/29	Capsa et al., 2022 [30]
54	Yes	No	ABC	CD45+, CD20+, BCL2+, MUM1+, BCL6+	Exophytic mass /9 × 8 × 8 cm	Normal	Biopsy	Surgery (radical trachelectomy in patient with previous subtotal hysterectomy + BSO) + CT (R- CHOP ×6 + RT (20 Gy)	DF/24	Akkour et al., 2021 [31]
52	Yes	No	GCB	CD20+ CD10+B CL-6-	Cervical mass/6 cm	-	Biopsy	CT (R-CHOP ×6) + RT (45Gy)	DF/18	Goda et al., 2020 [20]
50	Yes	No	GCB	CD20+ CD10+ BCL-6-	Cervical mass/3 cm	-	Biopsy	CT (R-CHOP ×6) + RT (45Gy)	DF/43	
39	No	No	ABC	CD20+ CD10- BCL-6-	Bulky cervical mass extending to upper third of vagina and lower uterine segment/8 cm	-	Biopsy	CT (R-CHOP ×6) + RT (45Gy)	DF/8	
36	No	No	-	LCA+ CD20+	Cervical mass/4 cm	-	Biopsy	CT (CHOP ×6)	DF/73	Gui et al., 2019 [32]
60	Yes	No	-	-	-	-	-	CT (R-CHOP ×6) + RT	DF/-	Boussios et al., 2018 [33]
55	Yes	No	-	-	Bulky endocervical mass/10 cm	-	Biopsy, D&C	CT (R-CHOP ×3) + LAVH	DF/36	Roberts et al., 2018 [34]
51	Yes	No	GCB	CD20+, CD5+, BCL2+, BCL6+, CD45, CD23, CD43, CD10- , EBER-, Cyclin D1-	Bulky exophytic cervical mass/10 cm	Normal	Biopsy	CT (R-CHOP ×6)	DF/24	Cubo et al.2017 [35]
39	No	No	Spindle cell or sarcomatoi d variant, GCB	LCA+, CD20+, CD45+, BCL6+,	Bulky endocervical mass/-	-	Biopsy	Surgery (TH) + CT (R- CHOP ×3)	DF/48	Fratoni et al., 2016 [36]
27	No	No	ABC	CD20+ MUM1+	Mass between uterus and bladder/6.5 × 2.3 cm	LSIL HPV -	LLETZ	CT (R-CHOP ×6)	DF/49	Posfai et al., 2015 [51]
20	No	No	-	-	-	-	Biopsy	CT (CHOP ×7)	DF/84	Cao et 2014 [37] al.
58	Yes	No	-	-	Bulky mass/-	-	Biopsy	CT (CHOP x6) + RT	CR + CNS relapse/47 alive/56	
49	No	No	-	CD20+ CD45+	Bulky exophitic cervical mass/	-	Biopsy	CT (CHOP ×6) + RT (46Gy)	DF/192	Mouhajir et al., 2014 [38]
85	Yes	No	-	CD20+	Bulky, exophytic cervical mass/7 × 4 cm	-	Biopsy	CT (R-CHOP ×3)	PR, refused other treatments /5	Binesh et al., 2012 [39]
54	Yes	No	-	LCA+ CD20+ BCL2 -	Friable cervical mass extending to low uterine segment/-	-	Biopsy	CT (R-CHOP ×6) + RT (35Gy)	DF/17	Parnis et al., 2012 [40]
52	Yes	No	-	CD20+ CD45+ CD3-	endocervical polyp /5 × 3 cm	Normal	Surgical specimen	Surgery (TH BSO)	DF/20	Vasudev et al., 2012 [41]
21	No	No	-	LCA+ CD45+ CD20+ CD79A+	Enlarged cervix/-	Normal	Biopsy	CT (R-CHOP ×6) + previous cryopreservation of embryos + ovarian suppression	DF/72	Parva et al., 2011 [42]
49	-	No	-	CD20+	Enlarged cervix/8 × 4.5 × 6 cm	-	Biopsy	CT (R-CHOP ×6) + RT (30 Gy)	DF/20	Upanal et al., 2011 [43]
44	No	No	-	LCA+, CD20+	Cervical mass/7 × 7 cm	-	Biopsy	CT (R-CHOP ×3) + RT (46Gy)	DF/15	Baijal et al., 2009 [5]
43	No	No	-	L26/CD20+	Bulky exophytic cervical mass/5 × 7.8 × 3 cm	Normal	Biopsy	CT (CHOP ×6)	DF/-	Ab Hamid et al., 2008 [9]
29	No	No	-	CD20+ CD79a+ BCL6+ CD30- CD3- CD10-	Cervical mass infiltrating upper vagina/3 cm	Normal	Biopsy	Ovarian transposition + CT (CHOP ×6) + RT (30Gy)	DF/54	Ferreri et al., 2008 [44]
60	Yes	No	-	CD20+ CD3+ CD5+ CD21+ CD23+ P53+ MUM1+	Cervical mass extending to upper vagina and left parametrium, bilateral hydronephrosis/7 cm	-	Biopsy	CT (CHOP ×6)	DF/-	Wuntakal et al., 2008 [45]
29	No	No	-	CD20+ LCA+ CD30- CD45-	Bulky cervical mass extending to upper vagina/5 cm	Normal	Biopsy	CT (CHOP ×3) + surgery (cold-knife conization) + CT (CHOP ×3)	DF/48	Lorusso et al., 2007 [52]
32	No	-	-	-	-	-	-	CT (CHOP ×6)	DF/91	Signorelli et al., 2007 [53]
45	-	-	-	-	Cervical mass/6 × 5 cm	-	-	CT (CHOP ×6) + surgery (TH + BSO + pelvic nodal sampling)	DF/38	
58	-	-	-	-	Cervical mass/2 × 1 cm	-	-	Surgery (TH + BSO) + CT (CHOP ×4)	DF/228	
54	-	-	-	-	-	-	-	Surgery (TH + BSO)	DF/118	
47	No	No	-	LCA+ CD20+	Cervical mass extending to parametrium bilaterally, uterus, upper vagina and with bilateral vesicoureteral junction obstruction/-	-	Biopsy	CT (CHOP ×3 + COP ×3 with Adriamicin omitted because of cardiotoxicity ) + RT (45Gy)	DF/13	Hariprasad et al., 2006 [46]
80	Yes	No	-	-	Necrotic, bleeding cervical growth extending to upper vagina, left parametrium and uterus/-	-	Biopsy	CT (CHOP ×6), RT omitted because of old age	DF/12	
43	Yes	-	-	CD20+ CD45+ BCL6+ BCL2- CD30- CD3-	Bulky cervical mass mimicking leiomyoma/10 cm	Normal	Surgical specimen	Surgery (TH) + CT (CHOP ×6)	DF/10	Semczuk et al. 2006 [47]
26	No	No	-	CD20+ CD3+ CD30+	Cervical mass/6.5 cm	-	Surgical specimen	Surgery (TH) + CT (R- CHOP)	DF/12	Gonzalez- Cejudo et al., 2006 [48]
51	-	No	-	LCA+, CD20+	Cervical mass/4 cm	HSIL	LEEP	Surgery (TH + BSO + pelvic and paraaortic LMP) + CT (CHOP ×6)	DF/19	Dursun et al., 2005 [54]
76	Yes	No	-	-	Cervical mass/3 cm	ASCUS	Biopsy	Surgery (TH + BSO + pelvic limphadenect	DF/14	Chan et al., 2005 [21]
67	Yes	No	-	-	Cervical polyp	-	Biopsy (polypectom y)	Surgery (TH + BSO + pelvic nodes dissection) + RT (44 Gy)	DF/120	Lee et al., 1998 [49]
65	Yes	No	-	-	Bulky exophytic cervical mass/-	-	Biopsy	Surgery (TH + BSO + pelvic nodes dissection) + RT (44 Gy)	DF/120	
50	Yes	No	-	CD45+	Firm multiple nodules involving cervix and upper vagina/10 cm	-	Biopsy	CT (CHOP ×4) + RT (46Gy) + CT (CHOP ×2)	DF/17	Chandy et al., 1998 [50]
64	Yes	No	-	LCA+ L26+ MB1+	Hards cervical mass/10 cm	-	Biopsy	CT (THP- COP x10)	DF/18	Nasu et al., 1998 [55]
37	No	No	Centroblast ic (Kiel classificati on)	-	Exophitic cervical mass extending to upper vagina and left parametrium	-	-	CT (cyclophospham ide, vincristine, prednisone ×3) + RT (45Gy)	DF/96	Makarewicz et al., 1995 [57]
33	No	No	Centroblast ic centrocytic	-	Cervical mass extending to upper third of vagina/-	Normal 1 year before	Surgical specimen	Surgery (TH+BSO+pelvi c limph node dissection)	Relapse after 6 months with a 6 cm tumour of upper vagina treated with CT (CHOP ×6), after it DF/42	
53	Yes	No	-	-	Cervical mass extending to upper vagina/ 4 × 10 cm	-	Biopsy	CT (CHOP + bleomycin) + RT (40 Gy) + CT (as previously)	DF/173	Stroh et al 1995 [10]
64	Yes	No	-	-	Cervical mass/8 Cm	-	Biopsy	RT (60 Gy) + CT (CHOP + bleomycin) + RT + CT	DF/165	
66	Yes	No	-	-	Cervical mass/3 Cm	-	Biopsy	CT (CHOP) + RT (40 Gy) + CT	DF/60	
67	Yes	No	-	-	Cervical mass/-	-	Biopsy	CT (ASAP, MBACOS, MINE) + RT (40 Gy)	PR/18	

CT: chemotherapy; proMECE/CytaBOM: cyclophosphamide, epirubicin, etoposide, prednisone, cytarabine, vincristine, bleomycin, and methotrexate); NK: natural killer; MALT: mucosa associated lymphoid tissue; RT: radiotherapy; DLBCL: diffuse large B-cell lymphoma; GC: germinal center; TH: total hysterectomy; STAH: subtotal abdominal hysterectomy; BSO: bilateral salpingo-oophorectomy; CHOP: cyclophosphamide, hydroxydaunorubicin, vincristine, prednisone; DF: disease free; LMP: lymphadenectomy; LAVH: laparoscopic assisted vaginal hysterectomy; R-CEOP: Rituximab + Cyclophosphamide, Etoposide, Prednisolone, Vincristine; ASAP: doxorubicin, solumedrol, ara-c, cisplatin; MBACOS: methotrexate, bleomycin, doxorubicin, cyclophosphamide, vincristine, solumedrol; MINE: Mesna, ifosfamide, novantrone, etoposide; GCB: germinal center type B-cell lymphoma); ABC: activated B-cell type lymphoma; PR: partial response; GCB: germinal center B-type; THP-COP: pirarubicin, cyclophosphamide, vincristine sulfate, prednisolone; ALK: Anaplastic lymphoma kinase; LLETZ: large loop excision of the transformation zone.

**Table 4:** Pregnancy after treatment.

Reference	Age	Treatment	Timing Post-Treatment	Mode of Delivery	Complications
Quaresima et al., 2022 [29]	30	CHOP + Rituximab + ovarian suppression (Leuprolide)	1 year	Induction of labour at 41 gw—Vaginal delivery	None
Posfai et al., 2015 [37]	27	CHOP + Rituximab	14 months	-	Miscarriage 8th gw
Parva et al., 2011 [43]	21	CHOP + Rituximab	5.5 years	Vaginal delivery	None
Ferreri et al., 2008 [44]	29	CHOP + Rituximab + (ovarian transposition) + RT	3 years	Vaginal delivery	None
Lorusso et al., 2007 [52]	29	CHOP + cold knife conization	3 years	Planned CS at 38 gw	None

Legend: CHOP: cyclophosphamide, hydroxydaunorubicin, vincristine, prednisone, GW: gestational weeks; CS: Caesarean section.

## Data Availability

The authors confirm that the data supporting the findings of this study are available within the article.

## Appendix A. JBI Checklist for Case Report and Case Series

**Table A1:** JBI Critical Appraisal Checklist for case reports.

D1.	Were patient’s demographic characteristics clearly described?
D2.	Was the patient’s history clearly described and presented as a timeline?
D3.	Was the current clinical condition of the patient on presentation clearly described?
D4.	Were diagnostic tests or assessment methods and the results clearly described?
D5.	Was the intervention(s) or treatment procedure(s) clearly described?
D6.	Was the post-intervention clinical condition clearly described?
D7.	Were adverse events (harms) or unanticipated events identified and described?
D8.	Does the case report provide takeaway lessons?

**Table A2:** JBI Critical Appraisal Checklist for case reports.

D1.	Were there clear criteria for inclusion in the case series?
D2.	Was the condition measured in a standard, reliable way for all participants included in the case series?
D3.	Were valid methods used for identification of the condition for all participants included in the case series?
D4.	Did the case series have consecutive inclusion of participants?
D5.	Did the case series have complete inclusion of participants?
D6.	Was there clear reporting of the demographics of the participants in the study?
D7.	Was there clear reporting of clinical information of the participants?
D8.	Were the outcomes or follow up results of cases clearly reported?
D9.	Was there clear reporting of the presenting site(s)/clinic(s) demographic information?
D10.	Was statistical analysis appropriate?
